# Caspase-1 Abrogates the Salutary Effects of Hypertrophic Preconditioning in Pressure Overload Hearts via IL-1β and IL-18

**DOI:** 10.3389/fmolb.2021.641585

**Published:** 2021-03-24

**Authors:** Fangjie Dai, Xuan Li, Xia Li, Zhiwen Ding, Ran Xu, Peipei Yin, Shijun Wang, Junbo Ge, Jian Wu, Yunzeng Zou

**Affiliations:** ^1^ Shanghai Institute of Cardiovascular Diseases, Zhongshan Hospital and Institutes of Biomedical Sciences, Fudan University, Shanghai, China; ^2^ School of Basic Medical Sciences, Fudan University, Shanghai, China

**Keywords:** cardiac hypertrophy, hypertrophic preconditioning, inflammation, caspase-1, MAPK

## Abstract

Cardiac hypertrophic preconditioning (HP) signifies cardioprotection induced by transient pressure overload to resist hypertrophic effects of subsequently sustained pressure overload. Although it is recently found that inflammation triggers the development of nonischemic cardiomyopathy, whether inflammation plays a role in the antecedent protective effects of HP remains unknown. Caspase-1 is a critical proinflammatory caspase that also induces pyroptosis; thus, we investigated the role of caspase-1 using a unique model of HP in mice subjected longitudinally to 3 days of transverse aortic constriction (TAC 3d), 4 days of de-constriction (De-TAC 4d), and 4 weeks of Re-TAC (Re-TAC 4W). Echocardiography, hemodynamics, histology, PCR, and western blot confirmed preserved cardiac function, alleviated myocardial hypertrophy and fibrosis, and less activated hypertrophic signaling effectors in Re-TAC 4W mice, compared with TAC 4W mice. Mechanistically, caspase-1 and its downstream targets IL-1β and IL-18, but not GSDMD, were less activated in Re-TAC 4W mice. Furthermore, in HP mice with AAV-9-mediated cardiac-specific caspase-1 overexpression, the salutary effects of HP were remarkably abrogated, as evidenced by exacerbated cardiac remodeling, dysfunction, and activation of IL-1β and IL-18. Collectively, this study revealed a previously unrecognized involvement of caspase-1 in cardiac HP by regulation of IL-1β and IL-18 and shed light on caspase-1 as an antecedent indicator and target for cardiac hypertrophy.

## Introduction

Pathological cardiac hypertrophy is a compensatory response of the heart challenged with pathological stimuli like pressure overload, valve stenosis, or regurgitation (Nakamura and Sadoshima, 2018; Wu et al., 2020a). While these stimuli persist, cardiac hypertrophy will eventually develop into heart failure (Oka et al., 2014; Li et al., 2018). Therefore, it is of great importance to explore the endogenous protective pathways to regress heart failure from the onset of cardiac hypertrophy. In recent years, the cardioprotective effect of hypertrophic preconditioning (HP), which is similar to that of ischemic preconditioning, has been confirmed by convincing evidence (Wei et al., 2015; Huang et al., 2017; Wu et al., 2020b). HP is induced by withdrawal of preset pressure overload to show resistance to subsequent pressure overload (Heusch, 2015; Wei et al., 2015). The HP phenomenon is expected to explain individual differences in cardiac hypertrophy (Yang et al., 2007; Cacciapuoti, 2011). For instance, the morbidity of cardiac hypertrophy in patients with hypertension is <50%, suggesting the involvement of HP to resist prohypertrophic stimulation in many patients (Cuspidi et al., 2012). However, although HP can help develop new therapy to intervene with cardiac hypertrophy early, its underlying mechanism remains elusive.

Growing evidence shows that cardiac inflammation can occur under nonischemic conditions such as cardiac hypertrophy and heart failure (Suetomi et al., 2018; Gong et al., 2020). Caspase-1 (also termed interleukin-converting enzyme) is a key member of proinflammatory caspases. After stimulation with pathogen-associated molecular patterns (PAMPs) or damage-associated molecular patterns (DAMPs), pro-caspase-1 together with NOD-like receptor 3 (NLRP3), and apoptosis-associated speck-like protein containing a CARD (ASC) can form inflammasome, resulting in the automatic cleavage of pro-caspase-1 to active caspase-1, which leads to activation of downstream proinflammatory cytokines IL-1β and IL-18, and cleavage of gasdermin D (GSDMD) NT terminal to induce the formation of plasma membrane pores, followed by inflammatory factors secretion and pyroptosis (Shi et al., 2015; Liu et al., 2018). Cardiac inflammation-related pyroptosis has been extensively studied in cardiac infarction, atherosclerosis, and ischemia-reperfusion, but similar studies on cardiac hypertrophy are still in paucity (Mezzaroma et al., 2011; Grebe et al., 2018; Toldo et al., 2018). Moreover, although it is recently found that caspase-1 is activated in mice after aortic banding and regulates angiotensin II-induced cardiac hypertrophy by cleavage of IL-1β (Bai et al., 2018; Suetomi et al., 2018), whether caspase-1 plays a role in HP requires further investigation. We thus designed this study to answer whether caspase-1 is involved in HP by regulation of IL-1β, IL-18, and GSDMD.

## Materials and Methods

### Mouse Models of TAC and HP

The animal study was reviewed and approved by the Animal Care and Use Committee of Zhongshan Hospital, Fudan University, and was in strict accordance with the National Institutes of Health Guide for the Care and Use of Laboratory Animals (revised in 1996).

Male C57BL/6J mice (8–10 weeks old, 20–25 g/mouse) were purchased from Shanghai SLAC Laboratory Animal Company. Pressure overload was induced by transverse aortic constriction (TAC), as we described previously (Wu et al., 2012). Briefly, mice were anesthetized with 2% isoflurane, intubated through the trachea, and connected to a ventilator. Thoracotomy was performed in the left parasternal second intercostal space to separate the aortic arch. Subsequently, the 6-0 silk thread was passed below the aortic arch, followed by ligation of the separated aortic arch with 27 G needle, which was carefully pulled out to result in annular constriction of ascending aorta. Then, the muscle and skin of mice were sutured with 4-0 silk thread and disinfected.

HP model was established as we described previously with appropriate modification to reduce mortality rate during operation (Huang et al., 2017; Wu et al., 2020b). Briefly, after the 1st TAC, a long thread was buried under the chest skin. Three days after TAC, a second thoracotomy was performed, the banding knot was relieved (De-TAC) by microforceps, and the same thread was buried under the chest skin again. Four days after De-TAC, a third thoracotomy was performed and the aortic arch was rebanded (Re-TAC) using the same thread during TAC and De-TAC (Supplementary Figure S1). In order to avoid the interference caused by repeated thoracotomy, mice in the sham group and TAC group also underwent thoracotomy three times at corresponding time points, while the aortic arch in the TAC group was only banded during the third thoracotomy.

### Echocardiography

The echocardiography was evaluated by a Vevo 2100 high-frequency ultrasound system with a 30 MHz scanner (VisualSonics, Toronto, ON, Canada). Under the anesthesia of 1.5% isoflurane, the left ventricular structure and function were evaluated in M-mode of the parasternal long-axis view (Wu et al., 2010; You et al., 2012; Huang et al., 2017). The indices included the following: heart rate (HR), left ventricular posterior wall end-diastolic thickness (LVPWTd), left ventricular posterior wall end-systolic thickness (LVPWTs), left ventricular ejection fraction (LVEF), and left ventricular fractional shortening (LVFS). Then, the probe was placed on the right side of the mouse and tilted horizontally to display the aortic arch section, and the peak systolic velocity of aortic arch flow (PSVa) was obtained (Huang et al., 2017)

### Invasive Hemodynamics

Left ventricular hemodynamics was assessed using the Power Laboratory system (AD Instruments, Castle Hill, NSW, Australia) connected to a micromanometric catheter (Millar 1.4-Fr, SPR 835, Millar Instruments, Houston, TX). After anesthesia with 1.5% isoflurane, the neck skin was cut off, and the right common carotid artery was isolated. The catheter was advanced into the left ventricle through the right common carotid artery. Left ventricular end-systolic pressure (LVESP) and left ventricular end-diastolic pressure (LVEDP) were acquired for left ventricular blood pressure evaluation; then, the maximal rate of pressure rising (Max dP/dt) and maximal rate of pressure fall (Min dP/dt) were collected for left ventricular systolic and diastolic function evaluation (Huang et al., 2017).

### Histological Analysis

After the hemodynamic study, the mice were killed by cervical dislocation. Then, the heart was removed, dried on filter paper, and weighed. The heart weight/body weight (HW/BW) was calculated. Subsequently, the heart was fixed under 4% paraformaldehyde and embedded in paraffin. And the papillary muscle at the most expansive part of the heart was sectioned along the short axis of the horizontal direction with the standard thickness of 4 μm. Hematoxylin-eosin (HE) staining was used to observe the morphology of cardiomyocytes. For visual evaluation of cardiac hypertrophy, the Texas Red™ X-labeled wheat germ lectin (WGA, Invitrogen Corp) was further used to observe cell membranes, and the nuclei were labeled with 4′,6-diamidino-2-phenylindole (DAPI). Masson’s trichrome staining was used to observe the degree of cardiac interstitial and perivascular fibrosis. For the cross-sectional area (CSA) and fibrosis measurements, the images were quantified by an image analysis system (ImageJ 1.52v National Institutes of Health, Bethesda, United States).

### Virus Infection

The myocardial specific caspase-1 was overexpressed *in vivo* by using an adeno-associated virus type 9 (AAV9) vector (pAOV-cTNT-EGFP-2A-Casp1-3Flag, Obio Biotechnology, Shanghai, China). A total of 1 × 10^11^ viral particles (vp) of AAV9-caspase-1 or vector virus AAV9-EGFP (pAOV-cTNT-EGFP-2A-MCS-3Flag, Obio Biotechnology, Shanghai, China) were injected into the tail vein of mice three weeks before surgery.

### Real-Time Quantitative Polymerase Chain Reaction (RT-qPCR)

Total RNA was extracted from heart tissue samples using TRIZOL reagent, and cDNA was synthesized by Reverse Transcription Kit (Takara, Kusatsu, Japan). RT-qPCR was carried out with SYBR Premix Ex Taq II (Takara, Kusatsu, Japan). Cardiac hypertrophy and fibrosis-related genes were amplified using the following primers: *ANP*, forward: GCT​TCC​AGG​CCA​TAT​TGG​AG and reverse: GGG​GGC​ATG​ACC​TCA​TCT​T; *BNP*, forward: GAG​GTC​ACT​CCT​ATC​CTC​TGG and reverse: GCC​ATT​TCC​TCC​GAC​TTT​TCT​C; *Col1a1*, forward: GGA​CGC​CAT​CAA​GGT​CTA​CTG​C and reverse: GAA​CGG​GAA​TCC​ATC​GGT​CAT; *Col3a1*, forward: CTC​AAG​AGT​GGA​GAA​TAC​TGG​GTT and reverse: GGT​ATG​TAA​TGT​TCT​GGG​AGG​C; *GAPDH*, forward: GCC​ATC​ACT​GCC​ACT​CAG​AA and reverse: GGC​ATG​TCA​GAT​CCA​CAA​CG. Expression levels of hypertrophic and fibrotic-related genes were shown as 2-ΔΔCt of the target gene relative to *GAPDH*.

### Western Blot (WB)

Total protein was obtained from the left ventricular heart tissue. Electrophoresis for protein (20–40 ug) was performed by 10% or 12.5% SDS-polyacrylamide gel and then protein was transferred to 0.22 μm PVDF membrane (Millipore, Billerica, United States). After blocking with 5% BSA for 2 h at room temperature, PVDF membrane with target protein was incubated with primary antibodies overnight at 4°C and secondary antibodies for 2 h at room temperature. The primary antibodies used for WB analysis were anti-p ERK1/2 (1:1,000; CST, United States) and anti-ERK1/2 antibody (1:1,000; CST, United States), anti-p p38 antibody (1:1,000; CST, United States), anti-p38 antibody (1:1,000; CST, United States), anti-p JNK antibody (1:1,000; CST, United States), anti-JNK antibody (1:1,000; CST, United States), anti-NLRP3 antibody (1:1,000; CST, United States), anti-caspase-1 antibody (1:500; Santa Cruz, United States), anti-cleaved caspase-1 antibody (1:1,000; CST, United States), anti-caspase-11 antibody (1:1,000; CST, United States), anti-GSDMD antibody (1:1,000; Abcam, United States), anti-IL-1β antibody (1:1,000; CST, United States), anti-IL-18 antibody (1:1,000; CST, United States), anti-cleaved IL-18 antibody (1:1,000; MBL, United States), and anti-GAPDH antibody (1:5,000; Abcam, United States). HRP-conjugated anti-rabbit or mouse secondary antibody was used as the secondary antibody (1:5,000; Weiao Biotechnology, Shanghai, China). WB detection system (Bio-Rad, United States) was used to detect the imprint after treatment of PVDF membrane by Millipore ECL kit (Invitrogen, Carlsbad, United States), and optical density was quantified using ImageJ analysis software (1.52v, National Institutes of Health, Bethesda, United States).

### Statistical Analysis

All continuous data in our study were presented as the mean ± standard error (SE). GraphPad Prism 8 (GraphPad Software, Version 8.01, San Diego, CA, United States) was used for statistical analysis. The normality tests were performed by the Shapiro–Wilk test. Based on the characteristics of data distribution, the difference between groups was analyzed by one-way ANOVA (only operation treatment) or two-way ANOVA (operation and virus treatments), followed by the Student–Newman–Keuls (SNK) test (data with normal distribution) or Kruskal–Wallis test followed by Dunn’s test (data not normally distributed) for multiple comparisons. A *p* value < 0.05 was considered statistically significant.

## Results

### HP Attenuates Cardiac Hypertrophy and Improves Function in Pressure Overload

**FIGURE 1:**
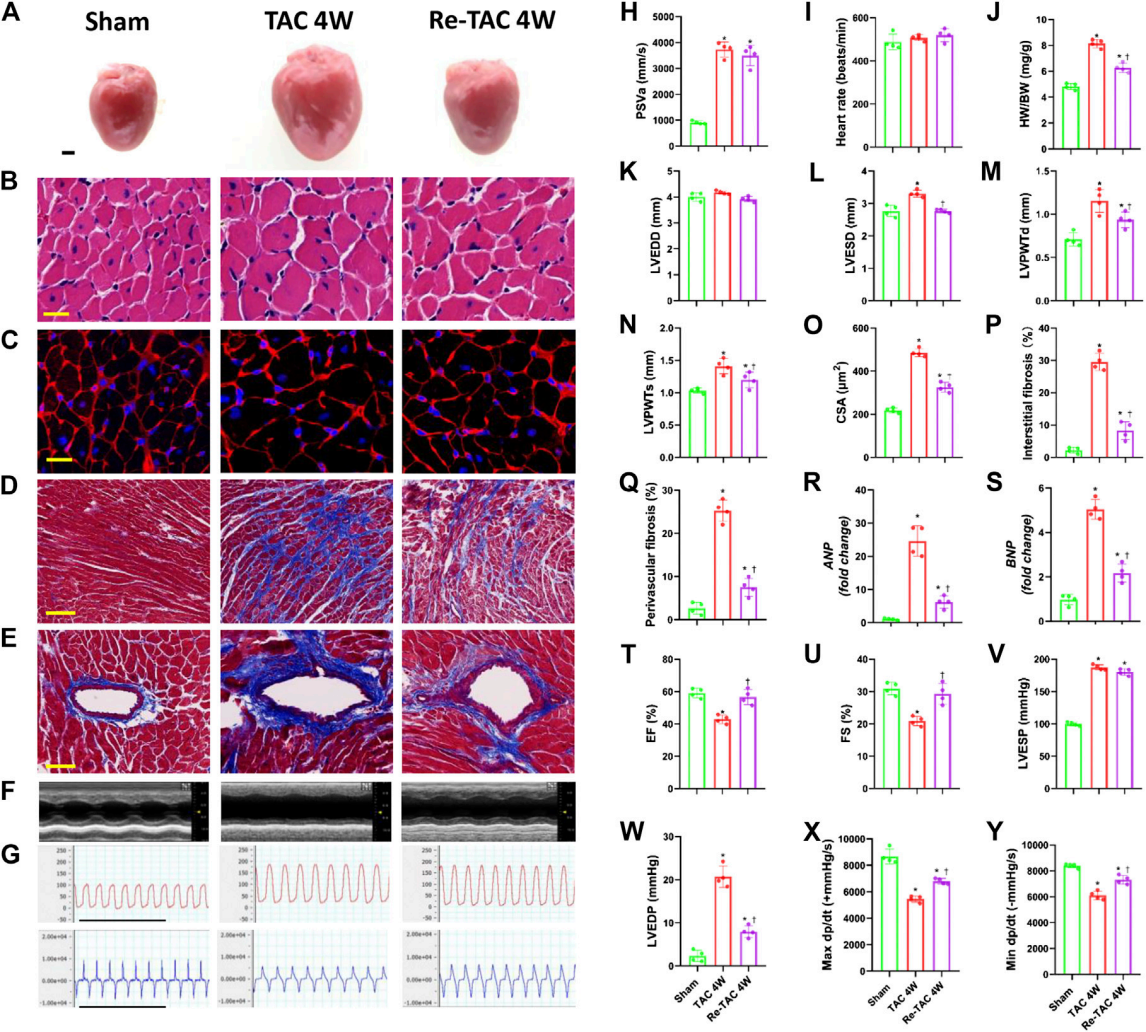
Hypertrophic preconditioning attenuates cardiac hypertrophy and improves cardiac dysfunction in pressure overload. **(A)** Gross appearance of the hearts. Scale bar, 1 mm. **(B)** Cardiomyocytes stained by HE. Scale bar, 20 μm. **(C)** Cardiomyocytes stained by WGA. Scale bar, 20 μm. **(D)** Interstitial fibrosis stained by Masson’s trichrome. Scale bar, 100 μm. **(E)** Perivascular fibrosis stained by Masson’s trichrome. Scale bar, 50 μm. **(F)** M-mode echocardiograms of left ventricles. **(G)** Left ventricular contraction and relaxation velocity images measured by a Millar catheter. Scale bar, 1s. **(H)** Peak systolic velocity of aortic arch flow (PSVa). **(I)** Heart rate. **(J)** Ratio of heart weight and body weight (HW/BW). **(K)** Left ventricular end-diastolic dimension (LVEDD). **(L)** Left ventricular end-systolic dimension (LVESD). **(M)** Left ventricular posterior wall end-diastolic thickness (LVPWTd). **(N)** Left ventricular posterior wall end-systolic thickness (LVPWTs). **(O)** CSA of cardiomyocytes. **(P)** Quantitative interstitial fibrosis. **(Q)** Quantitative perivascular fibrosis. **(R, S)** Real-time quantitative PCR analyses for the expression of *ANP* and *BNP*, respectively. **(T)** Left ventricular ejection fraction (LVEF). **(U)** Left ventricular fractional shortening (LVFS). **(V)** Left ventricular end-systolic pressure (LVESP). **(W)** Left ventricular end-diastolic pressure (LVEDP). **(X)** Maximal +dp/dt. **(Y)** Minimal -dp/dt. *n* = 4. *p < 0.05 vs. sham, ^†^*p* < 0.05 vs. TAC 4W, data analyzed by one-way ANOVA except for data in **(K)** LVEDD, which were analyzed by Kruskal–Wallis test for the data in the sham group were not normally distributed.

Both TAC 4W and Re-TAC 4W mice showed similar HRs and similarly elevated PSVa compared with those of sham mice (Figures 1H,I, Supplementary Figure S2). Compared with sham mice, TAC 4W mice showed prominent cardiac hypertrophy, as measured by increased HW/BW (Figure 1J), inner dimension (Figures 1K,L), LV wall thickness (Figures 1M,N), and fibrosis (Figures 1D,E,P–Q), enhanced levels of reprogramming of fetal genes *ANP* and *BNP* (Figures 1R,S), and augmentation of cardiomyocyte size (Figures 1A–C,O). Re-TAC 4W mice demonstrated that HP decreased the hypertrophic response compared with TAC 4W mice.

Meanwhile, TAC 4W mice showed marked cardiac dysfunction, as demonstrated by depressed LVEF and LVFS (Figures 1F,T,U), which was due to notable dilation of LVESD (Figure 1L). On the contrary, HP improved cardiac function and reduced LVESD to the level of sham mice. We also employed invasive hemodynamics to further assess the effects of HP on cardiac function. We found that LVESP increased in both the TAC group and HP group, while the HP group showed a significant reduction in LVEDP compared with that of the TAC group (Figures 1G,V,W). LV systolic and diastolic functions were also improved by HP, as indicated by higher maximal dP/dt and minimal dP/dt, respectively (Figures 1G,X,Y).

### HP Blunts the Signaling Networks of Hypertrophy and Fibrosis Induced by Pressure Overload

**FIGURE 2:**
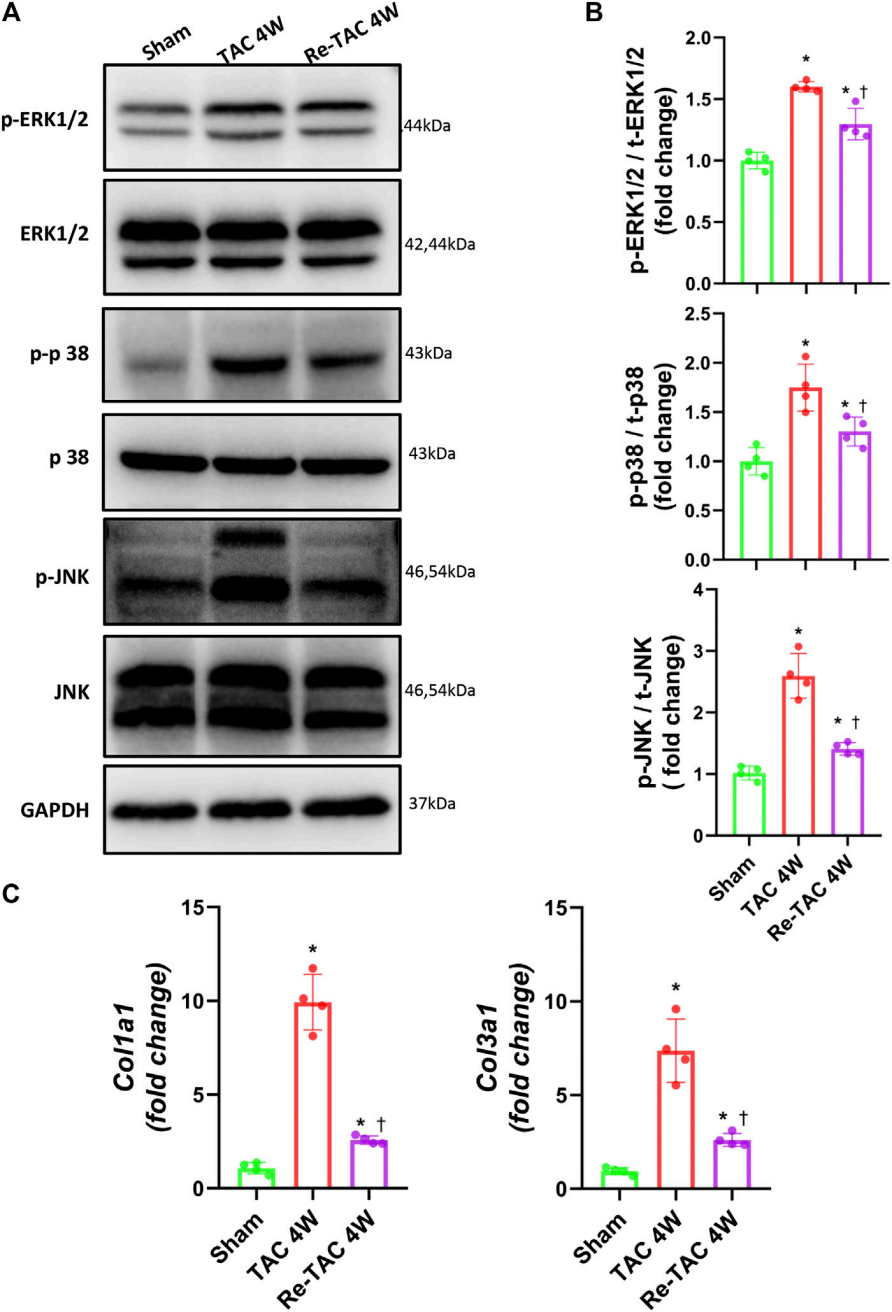
Hypertrophic preconditioning compromises signaling in hypertrophy and fibrosis induced by pressure overload. **(A)** Western blot analysis of ERK/p38/JNK signaling in sham, TAC, and Re-TAC hearts. **(B)** Quantification of ERK/p38/JNK signaling. **(C)** Real-time quantitative PCR analyses for the expression of fibrosis-related factors *Col1a1* and *Col3a1*. *n* = 4. *p < 0.05 vs. sham, ^†^*p* < 0.05 vs. TAC 4W, one-way ANOVA.

Mitogen-activated protein kinase (MAPK) is a type of protein kinase that is involved in directing cellular responses to hypertrophic stimuli (Zhang et al., 2017; You et al., 2018). The MAP kinases can be grouped into three main families, namely, ERK1/2 (extracellular-signal-regulated kinases), p38/SAPKs (stress-activated protein kinases), and JNK1/2 (Jun amino-terminal kinases). MAPK plays an important role in the development of cardiac hypertrophy (Li et al., 2016; Tamura et al., 2019). We, therefore, assessed the protein expression levels of ERK1/2, p38, and JNK. Compared with the sham group, the phosphorylation levels of ERK, p38, and JNK in both TAC and HP groups were significantly increased. Nevertheless, the hypertrophy signaling in Re-TAC 4W mice group was significantly downregulated compared with the TAC group (Figures 2A,B).

As for fibrosis signaling, compared with the sham group, fibrosis-related factors *COL1A1* and *COL3A1* were greatly elevated in TAC 4W mice, whereas they were much less elevated in Re-TAC 4W (Figure 2C).

### HP Mitigates Activation of Caspase-1 and Its Downstream Targets IL-1β and IL-18, but Not GSDMD

**FIGURE 3:**
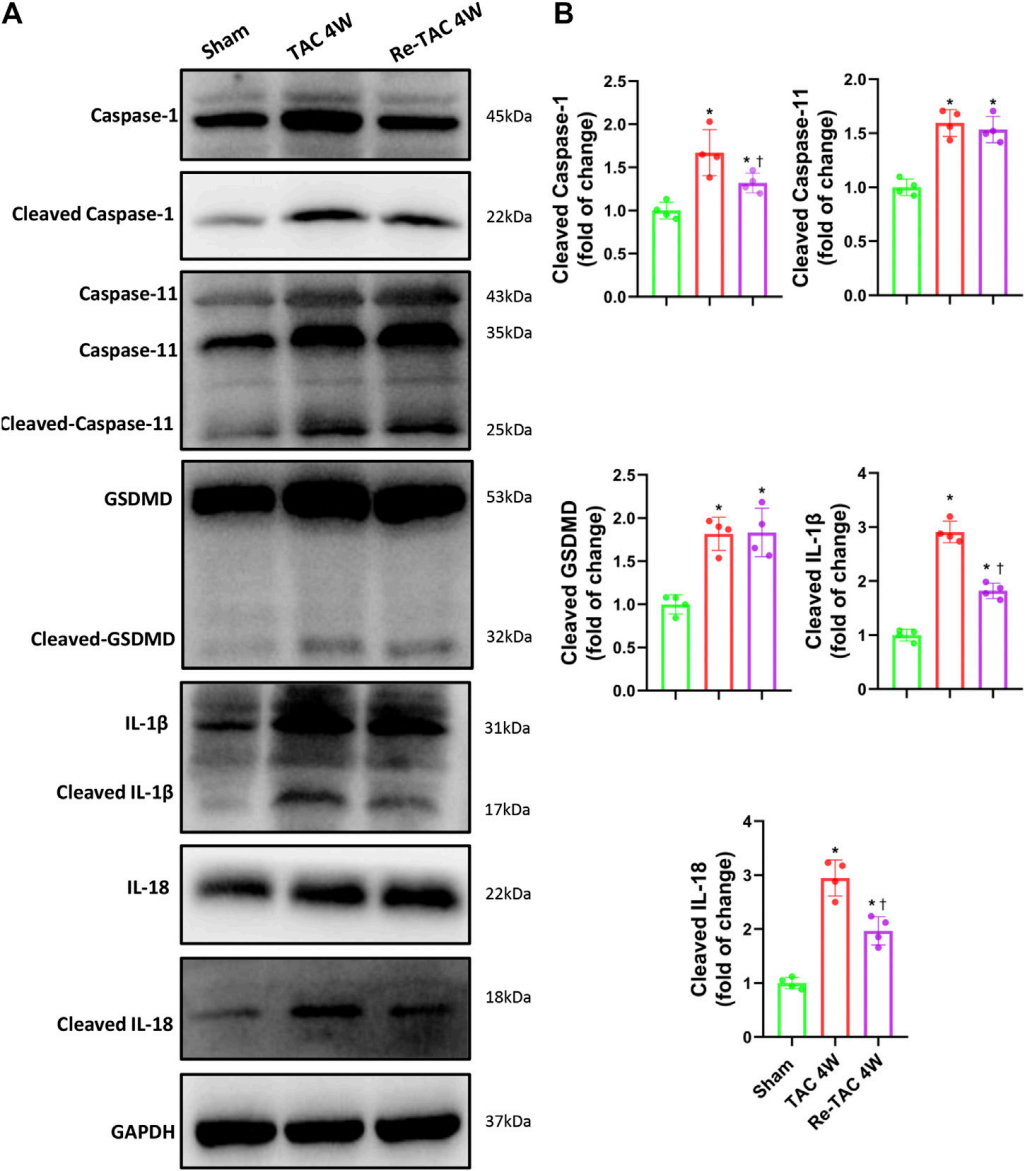
Hypertrophic preconditioning alleviates the activation of caspase-1 and its downstream targets IL-1β and IL-18, except GSDMD. **(A)** Western blot analysis of caspase-1, caspase-11, GSDMD, IL-1β, and IL-18 in sham, TAC, and Re-TAC hearts. **(B)** Quantification of cleaved caspase-1, cleaved caspase-11, cleaved GSDMD, cleaved IL-1β, and cleaved IL-18, relative to GAPDH. *n* = 4. *p < 0.05 vs. sham, ^†^*p* < 0.05 vs. TAC 4W, one-way ANOVA.

To unveil the changes of caspase-1 in HP, we found remarkable activation of caspase-1 in TAC 4W mice (Figures 3A,B). In contrast, Re-TAC 4W mice showed significantly decreased caspase-1 activation compared with TAC 4W mice (Figures 3A,B), suggesting close relations of caspase-1 with HP. Caspase-1 induces inflammation through cytokines IL-1β and IL-18 and causes pyroptosis through executor GSDMD (Shi et al., 2015). To further answer whether its downstream targets were also associated with HP, we found that IL-1β and IL-18 were both less activated in Re-TAC 4W mice, compared with TAC 4W mice (Figures 3A,B). Intriguingly, although compared with sham mice, the activation of GSDMD was increased in both TAC and Re-TAC mice, no significant difference was observed between the two groups (Figures 3A,B). Considering that caspase-1 and caspase-11 mediate a canonical pathway and a noncanonical pathway of pyroptosis, respectively, we also investigated the changes of caspase-11. We found caspase-11 was activated in TAC 4W mice but remained markedly activated in Re-TAC 4W mice, suggesting that the augmented pyroptosis originated in a noncanonical pathway (Figures 3A,B). To unveil the mechanism of the caspase-1 variations, we also investigated the change of NLRP3, the upstream effectors of caspase-1. In accordance with the caspase-1 level, the expression of NLRP3 was upregulated in TAC 4W mice but was downregulated in Re-TAC 4W mice (Supplementary Figure S3). Taken together, our findings indicated that HP mitigated the function of caspase-1 through attenuation of inflammation rather than pyroptosis.

### Overexpression of Caspase-1 Compromises the Protective Effects of HP on Cardiac Hypertrophy and Function

**FIGURE 4:**
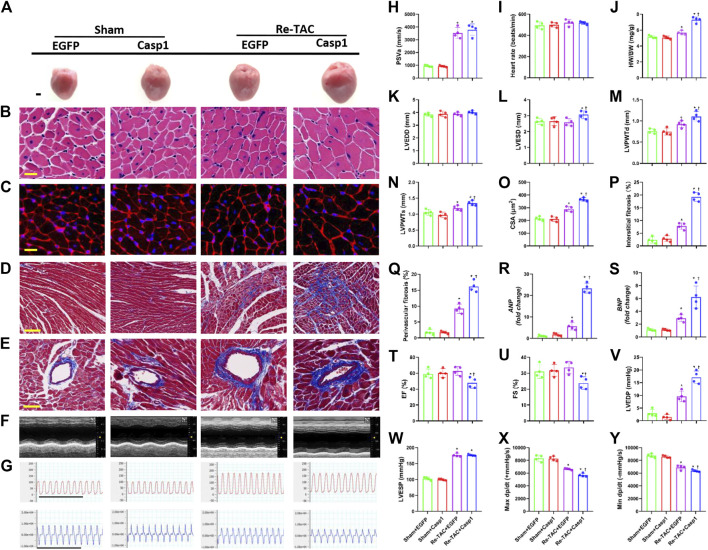
Overexpression of caspase-1 abrogates the salutary effects of hypertrophic preconditioning in pressure overload hearts. **(A)** Gross appearance of the hearts. Scale bar, 1 mm. **(B)** Cardiomyocytes stained by HE. Scale bar, 20 μm. **(C)**, Cardiomyocytes stained by WGA. Scale bar, 20 μm. **(D)** Interstitial fibrosis stained by Masson’s trichrome. Scale bar, 100 μm. **(E)** Perivascular fibrosis stained by Masson’s trichrome. Scale bar, 50 μm. **(F)**, M-mode echocardiograms of left ventricles. **(G)** Left ventricular contraction and relaxation velocity images measured by a Millar catheter. Scale bar, 1s. **(H)** Peak systolic velocity of aortic arch flow (PSVa). **(I)** Heart rate. **(J)** Ratio of heart weight and body weight (HW/BW). **(K)** Left ventricular end-diastolic dimension (LVEDD). **(L)** Left ventricular end-systolic dimension (LVESD). (**M**) Left ventricular posterior wall end-diastolic thickness (LVPWTd). **(N)** Left ventricular posterior wall end-systolic thickness (LVPWTs). **(O)** CSAs of cardiomyocytes. **(P)** Quantitative interstitial fibrosis. **(Q)** Quantitative perivascular fibrosis. **(R, S)** Real-time quantitative PCR analyses for the expression of *ANP* and *BNP*, respectively. **(T)** Left ventricular ejection fraction (LVEF). **(U)** Left ventricular fractional shortening (LVFS). **(V)** Left ventricular end-systolic pressure (LVESP). **(W)** Left ventricular end-diastolic pressure (LVEDP). **(X)** Maximal +dp/dt **(Y)**. Minimal -dp/dt. *n* = 4. *p < 0.05 vs. Sham+EGFP (Enhanced Green Fluorescent Protein, EGFP) or Sham+Casp1 (caspase-1 overexpression, Casp1), †*p* < 0.05 vs. Re-TAC+EGFP, two-way ANOVA.

To further clarify the relationship between caspase-1 and HP in the development of hypertrophy and fibrosis, we constructed an AAV9 virus vector to overexpress caspase-1 in mice hearts through tail vein injection. Overexpression of caspase-1 had no significant effect on PSVa and HRs (Figures 4H,I, Supplementary Figure S4). However, caspase-1 overexpression dramatically abrogated the beneficial effects of HP, which was evidenced by increased HW/BW (Figure 4J), inner dimension (Figures 4K,L), LV wall thickness (Figures 4M,N), and fibrosis (Figures 4D,E,P,Q), enhanced levels of *ANP* and *BNP* (Figures 4R,S), and augmentation of cardiomyocyte size (Figures 4A–C,O). Consistently, caspase-1 overexpression deteriorated cardiac function in HP mice, which was indicated by lower LVEF (Figures 4F,T), LVFS (Figures 4F,U), maximal dP/dt (Figures 4G,X), minimal dP/dt (Figures 4G,Y), and higher LVEDP (Figures 4G,V), while LVESP (Figures 4G,W) did not change significantly.

### Overexpression of Caspase-1 Diminishes the Protective Effect of HP on the Signaling Networks of Hypertrophy and Fibrosis

**FIGURE 5:**
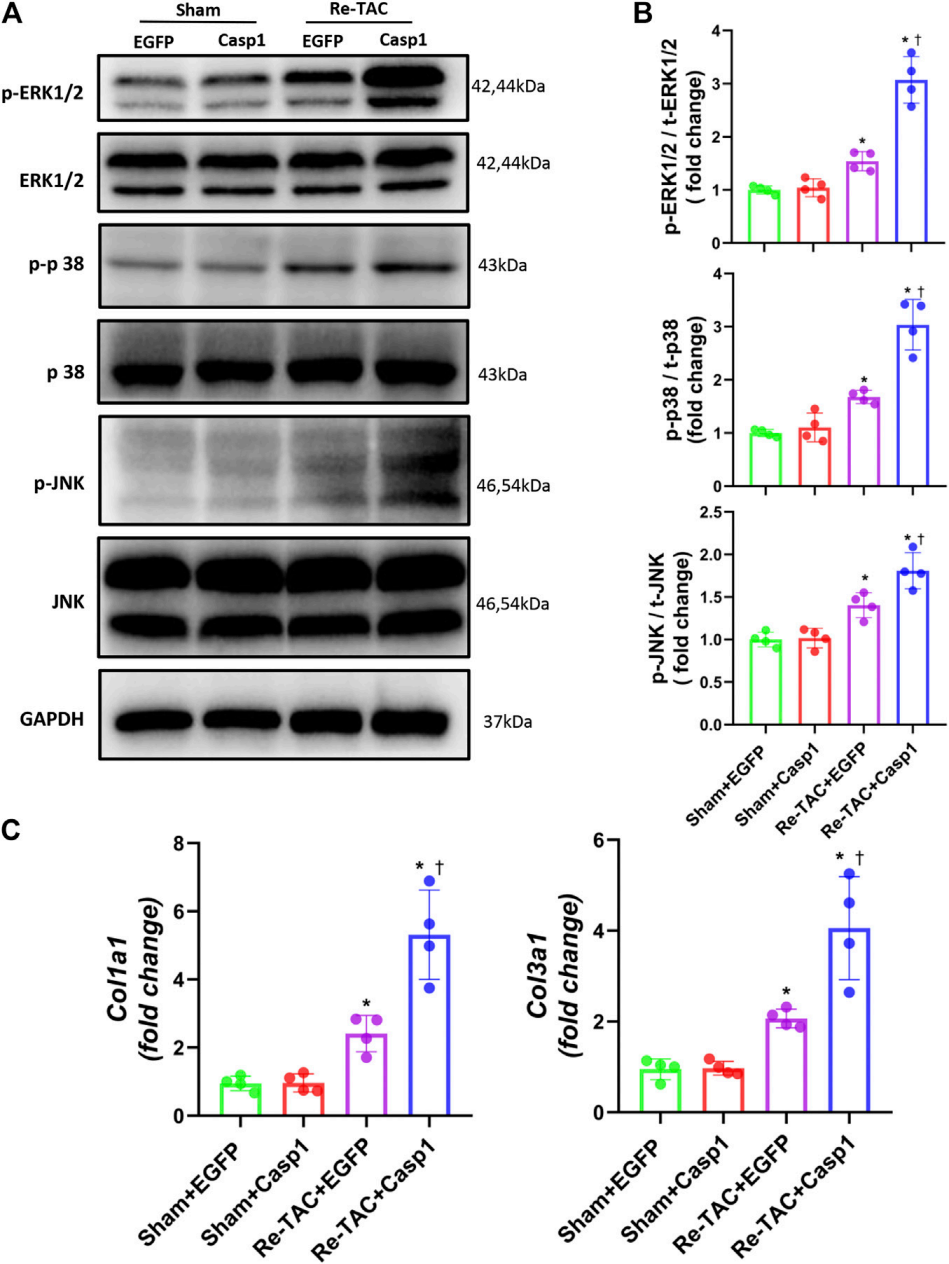
Overexpression of caspase-1 reverses the benefits of HP on the signaling networks of hypertrophy and fibrosis. **(A)** Western blot analysis of ERK/p38/JNK signaling in Sham+EGFP, Sham +Casp1, Re-TAC+EGFP, and Re-TAC+Casp1 hearts. **(B)** Quantification of ERK/p38/JNK signaling. **(C)** Real-time quantitative PCR analyses for the expression of fibrosis-related factors *Col1a1* and *Col3a1*. *n* = 4. *p < 0.05 vs. Sham+EGFP or Sham+Casp1, ^†^*p* < 0.05 vs. Re-TAC+EGFP, two-way ANOVA.

To answer whether caspase-1 alters the signaling pathways underlying the hypertrophic response in the context of HP, we examined the activation levels of MAPK under a Re-TAC background. Compared with EGFP treated Re-TAC mice, caspase-1 overexpression noticeably enhanced phosphorylated ERK, p38, and JNK (Figures 5A,B). In addition, caspase-1 overexpression dramatically aggravated the gene levels of *COL1A1* and *COL3A1* (Figure 5C).

### Overexpression of Caspase-1 Abolishes the Inflammation-Reducing Effect of HP by Aggravation of IL-1β and IL-18

**FIGURE 6:**
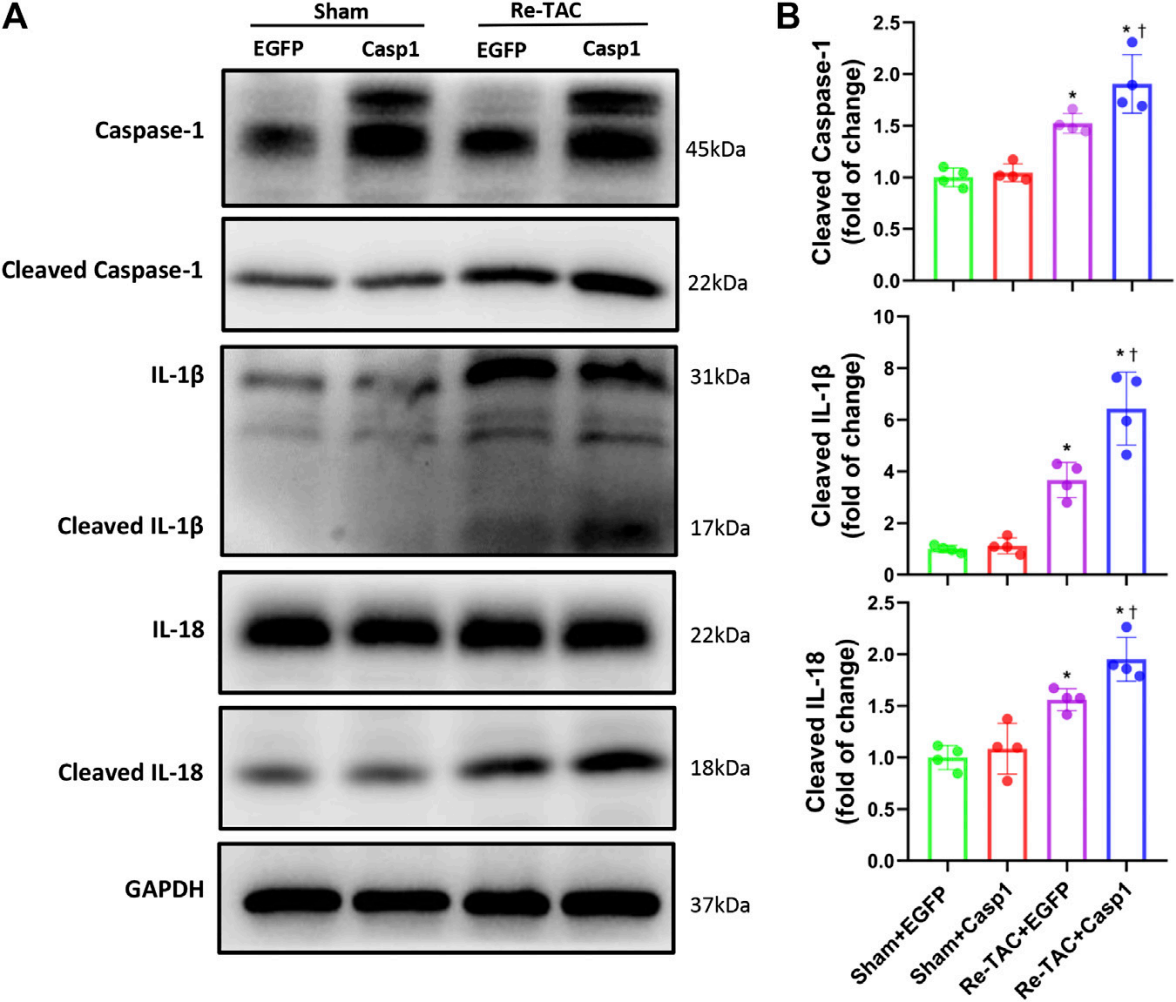
Overexpression of caspase-1 aggravates inflammatory activation of IL-1β and IL-18 in the HP. **(A)** Western blot analysis of caspase-1, IL-1β, and IL-18 in Sham+EGFP, Sham+Casp1, Re-TAC+EGFP, and Re-TAC+Casp1 hearts. **(B)** Quantification of cleaved caspase-1, cleaved IL-1β, and cleaved IL-18 relative to GAPDH. *n* = 4. *p < 0.05 vs. Sham+EGFP or Sham+Casp1, ^†^*p* < 0.05 vs. Re-TAC+EGFP, two-way ANOVA.

Since HP mitigates activation of caspase-1 mediated inflammatory cytokines IL-1β and IL-18, we examined whether IL-18 and IL-1β were involved in the detrimental effects of caspase-1 overexpression in HP mice. As expected, we found that caspase-1 overexpression aggravated the activation of IL-1β and IL-18 in Re-TAC 4W mice (Figures 6A,B), recapitulating that HP attenuates cardiac hypertrophy and regresses heart failure through downregulation of the inflammatory cascade of caspase-1.

## Discussion

In this study, we provide the first direct evidence that caspase-1 is involved in cardiac HP. The activation of caspase-1 was alleviated during HP, while the overexpression of caspase-1 greatly blunted the beneficial effects of HP through IL-1β- and IL-18-mediated aggravation of MAPK hypertrophic signaling pathways, suggesting caspase-1 serves as an antecedent indicator and predisposing factor of cardiac hypertrophy.

Pathological cardiac hypertrophy is an independent risk of heart failure, but the underlying mechanisms and early interventions remain largely unknown (Kurrelmeyer et al., 1998; Nakamura and Sadoshima, 2018; Wang et al., 2018; Wu et al., 2020a). In 2015, Wei et al. raised the concept of HP with solid evidence for the first time and concluded the benefits of HP through attenuation of cardiac hypertrophy and preservation of cardiac function (Wei et al., 2015). We and others also have indicated that HP is of great significance in delaying the progression of heart failure through regulation of several signaling effectors such as activation of S100A8/A9 and reduced ERK1/2 activation (Wei et al., 2015; Huang et al., 2017). Our current study first reported the important role of proinflammatory caspase in HP, which would help mechanistic and pharmaceutic studies on inflammation and the onset of nonischemic cardiomyopathy.

Chronic inflammatory response during sustained myocardial injury can lead to pathological cardiac hypertrophy and dysfunction, ultimately resulting in heart failure (Schirone et al., 2017; Bacmeister et al., 2019). Caspase-1, as a key member of the proinflammatory family of caspases, was known for mediating adverse cardiac remodeling in angiotensin II-induced cardiac hypertrophy and surgical myocardial infarction (Frantz et al., 2003; Bai et al., 2018). Although cardiac hypertrophy is observed in caspase-1 knockout mice subjected to renal ischemia/reperfusion (Trentin-Sonoda et al., 2019), caspase-1 is confirmed to induce inflammatory infiltration during long-term cardiac pressure overload (Suetomi et al., 2018). In HP, removal of short-term pressure overload mitigates caspase-1, suggesting the formation of “traumatic memory” at the early stage of HP, which might awaken endogenous protective mechanisms (such as downregulation of MAPK) when challenged with subsequent long-term pressure overload. In addition, after myocardium-specific overexpression of caspase-1, the cardioprotective effect of HP was abrogated. The results showed that caspase-1 effectively disrupted the endogenous protective effect formed by HP. Other studies are in line with our findings. It is reported that knockdown of caspase-1 attenuates the right ventricular remodeling induced by pulmonary hypertension in mice (Udjus et al., 2019), mitigates left ventricular dilation after myocardial infarction in mice (Frantz et al., 2003), and weakens angiotensin II-induced cardiomyocytes hypertrophy (Bai et al., 2018). It is also worth noting that the caspase-1 downstream inflammatory cytokines, IL-1β and IL-18, accelerate the transition from cardiac remodeling to heart failure (Sano et al., 2018; Xiao et al., 2018) and thus are expected to be potential drug targets for heart failure (Yoshida et al., 2014; Mocan et al., 2019). In this study, decreased IL-1β and IL-18 expression levels were evident in HP, indicating that the cardioprotective effect of HP is related to the downregulation of IL-1β and IL-18. Moreover, after the overexpression of caspase-1 in HP model, upregulation of IL-1β and IL-18 was observed, recapitulating the involvement of caspase-1-IL-1β/ caspase-1-IL-18 in HP.

Besides activation of downstream proinflammatory cytokines IL-1β and IL-18, caspase-1 also induces cleavage of GSDMD NT terminal to cause pyroptosis. Although pyroptosis has been well documented in cardiovascular diseases such as myocardial infarction and atherosclerosis (Zhaolin et al., 2019), we in the present study did not find significant changes in GSDMD between TAC and Re-TAC hearts. Considering that both caspases 1 and 11 cleave the 53 kDa inactive precursor form of GSDMD to generate an active GSDMD p30 fragment in a canonical and noncanonical pathway, respectively (Abe and Morrell, 2016), we also examined whether caspase-11 was changed by HP and found that the cleaved caspase-11 was not significantly different after HP treatment. Thus, we speculate that caspase-11 supplements GSDMD when the caspase-1 stimulation is reduced, making pyroptosis marginal in HP.

### Limitations

This study indicates that myocardium caspase-1 overexpression diminishes the benefits of HP in pressure overload mouse hearts. However, the heart is composed of cardiomyocytes, fibroblasts, endothelial cells, and immune cells, not only cardiomyocytes (Litviňuková et al., 2020). We did not differentiate the effects of HP and caspase-1 on cardiomyocytes from those on other types of cells, especially immune cells, nor did we investigate the communication between cardiomyocytes and other types of cells. With the application of state-of-the-art technology in cardiovascular research, such as transcriptome analysis and single-cell sequencing technology (Murashige et al., 2020; Wang et al., 2020), more mechanisms of HP will be explored in-depth.

## Conclusion

Our study provides the first evidence that caspase-1 abrogates the favorable effects of HP through IL-1β and IL-18, which is associated with the aggravation of MAPK hypertrophic signaling networks. Early intervention of caspase-1 may drive a promising strategy on regression of cardiac hypertrophy and heart failure.

## Data Availability

The datasets presented in this study can be found in online repositories. The names of the repository/repositories and accession number(s) can be found in the article/Supplementary Material.
