# Supplementary material for: Caspase-1 Abrogates the Salutary Effects of Hypertrophic Preconditioning in Pressure Overload Hearts via IL-1β and IL-18
Source: Front Mol Biosci. 2021 Mar 24;8:641585. doi: 10.3389/fmolb.2021.641585 (PMC8024560; doi:10.3389/fmolb.2021.641585)
Supplement: Supplementary file 1 [file table1.docx]

## Supplementary Figures


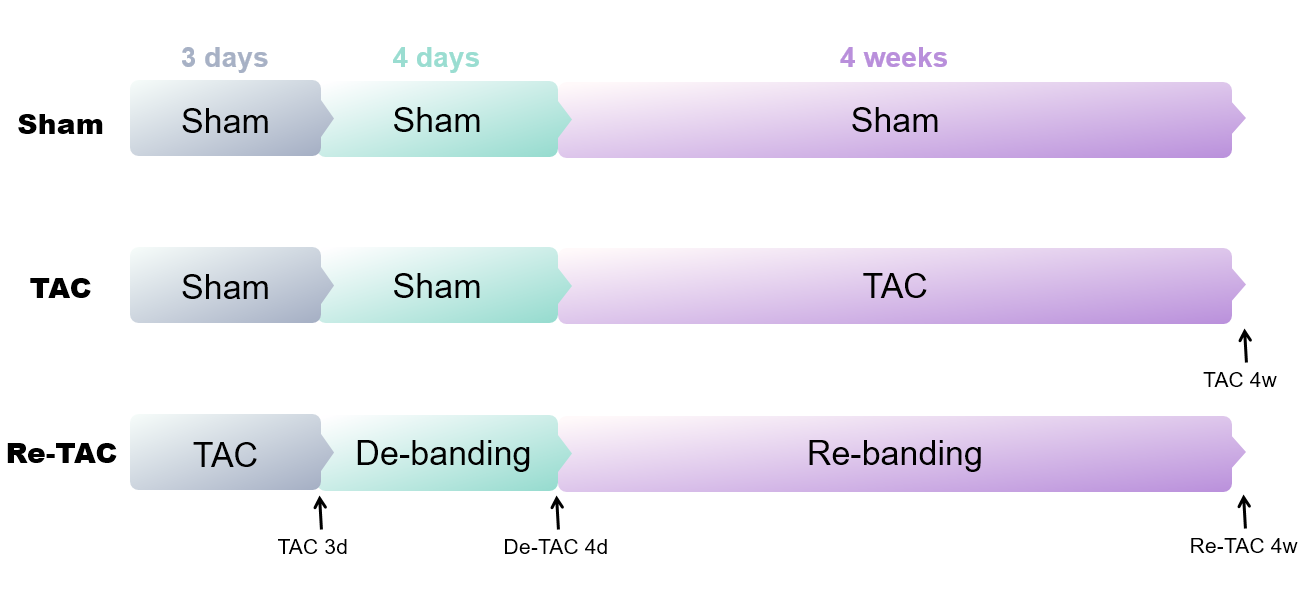


**Supplementary Figure 1.** Schematic of the process of mice operations. In Re-TAC group, mice were subjected to transverse aortic constriction for 3 days (TAC 3d). Subsequently the constrictions were relieved for 4 days (De-TAC 4d) and then these mice were subjected to TAC again for 4 weeks (Re-TAC 4w). Mice in sham group and TAC group also underwent three times of thoracotomy at corresponding time points, while the aortic arch in TAC group was only banded during the third thoracotomy. The cardioprotection of HP is induced by withdrawal of preset pressure overload (4 days of De-TAC after 3 days of TAC) and shows resistance to subsequent pressure overload (Re-TAC).

**
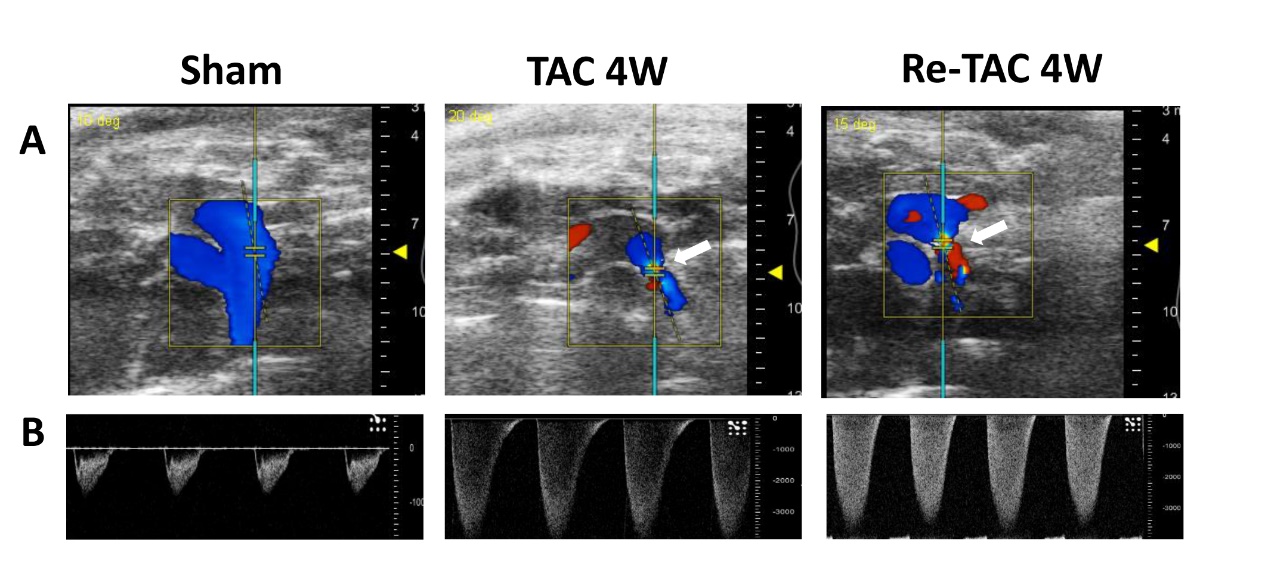
**

**Supplementary Figure 2**. The aortic banding site evaluation in TAC and HP model (**A**), Color doppler flow images of aortic arches. White arrow in the pane indicates the aortic banding site. (**B**), Pulsed wave doppler flow images of aortic arches.

**
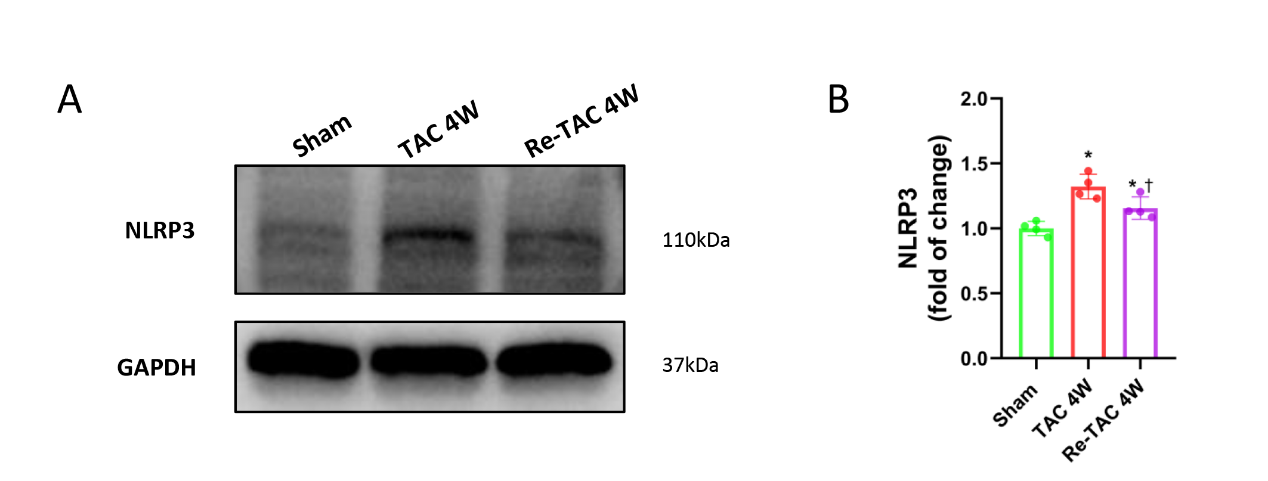
**

**Supplementary Figure 3.** Hypertrophic preconditioning alleviates the activation of NLRP3. (**A**), Western blot analysis of NLRP3 in Sham, TAC and Re-TAC hearts. (**B**), Quantification of NLRP3. n=4. * p < 0.05 vs. Sham, † p < 0.05 vs. TAC 4W, One-way ANOVA.

**
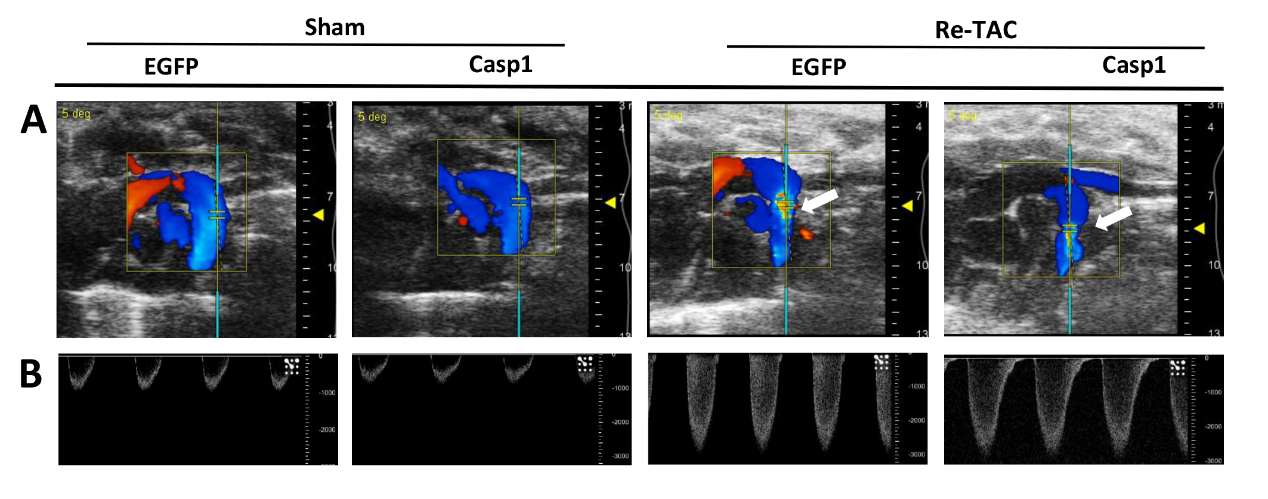
**

**Supplementary Figure 4**. The aortic banding site evaluation in HP model with AAV9 injection (**A**), Color doppler flow images of aortic arches. White arrow in the pane indicates the aortic banding site. (**B**), Pulsed wave doppler flow images of aortic arches.
