# Supplementary material for: Caspase-1 Abrogates the Salutary Effects of Hypertrophic Preconditioning in Pressure Overload Hearts via IL-1β and IL-18
Source: Front Mol Biosci. 2021 Mar 24;8:641585. doi: 10.3389/fmolb.2021.641585 (PMC8024560; doi:10.3389/fmolb.2021.641585)
Supplement: Supplementary file 2 [file table2.docx]

**Response to editors and reviewers**

Manuscript ID: 641585

Manuscript title: Caspase-1 abrogates the salutary effects of hypertrophic preconditioning in pressure overload hearts via IL-1β and IL-18

**Editor:**

***The interactive review of your manuscript "Caspase-1 abrogates the salutary effects of hypertrophic preconditioning in pressure overload hearts via IL-1β and IL-18" submitted to Frontiers in Molecular Biosciences, section Cellular Biochemistry has now been activated. The reviewers recommended that you make moderate amendments to your manuscript. Please respond within the next 17 days to all comments raised by the reviewers in the online review forum. You can also submit a revised version of your manuscript at that time. We encourage you to submit your documents with tracked changes to highlight the revisions. There can be more than one iteration between authors and reviewers, but only when all comments by each reviewer have been addressed successfully can the review be finalized.***

**Authors:** We sincerely thank the reviewers and editors for providing the opportunity of revising the manuscript. Their constructive comments greatly help us improve the manuscript. We have addressed all the comments and have revised our manuscript for your reconsideration, which is summarized below.

**Reviewer 1:**

**Please summarize the main findings of the study.**

***Reviewer 1:*** *The study focuses on the role of caspase-1 in a hypertrophic preconditioning (HP) murine model. The authors illustrated that the inflammatory caspase-1 and MAPK pathway are involved in the protective mechanism of HP. The authors also elegantly demonstrate the detrimental role of caspase-1 and its antecedent effects in the onset of hypertrophy, through gain-of-function manners, revealing the cardiac protective role of HP by downregulating caspase-1 and its downstream IL-1β and IL-18 activation.*

**Authors:** We appreciate the reviewer’s summarization of the main findings of the study.

**Please highlight the limitations and strengths.**

***Reviewer 1:*** *In general, this study is potentially interesting. The study is well designed, the methods, results and data interpretation support the conclusions. However, there are still some concerns needed to be addressed, please see Q5 for details.*

**Authors:** We thank the reviewer for the encouragement. We sincerely hope we have addressed the reviewer’s concerns, which can be found in detail in Q3.

**Please comment on the methods, results and data interpretation. If there are any objective errors, or if the conclusions are not supported, you should detail your concerns.**

***Reviewer 1:*** *1. The authors use a unique model of hypertrophic conditioning (HP) in mice subjected longitudinally to 3 days of transverse aortic constriction (TAC 3d), 4 days of de-constriction (De-TAC 4d), and 4 weeks of Re-TAC (Re-TAC 4W), which is somehow a complex process. It would be easily understood for* *future readers if there is a clear* *schematic of HP.*

**Authors:** We sincerely thank the reviewer's constructive suggestion for improving the manuscript. To clarify the complex process of HP and make a better understanding for the future readers, we have added a schematic of HP in our revised manuscript (please see Supplementary Figure 1).

*2. As for the mechanism of the inflammatory caspase-1, it is worthy of considering the changes its* *upstream effectors, such as the NLRP3 inflammasome.*

**Authors:** We thank the reviewer's pertinent suggestion for investigating the change of the upstream effectors of caspase-1. We detected the protein expression of NLRP3 and discovered it was upregulated in TAC 4W mice, and was downregulated in Re-TAC 4W mice, which was consistent with the change of caspase-1, recapitulating the caspase-1 mediated reduction of inflammatory level in the HP. We have supplemented these results in the revised manuscript (please see Supplementary Figure 3)

*3. For a better understanding of the myocardium specificity of AAV9 for readers, it should be emphasized in* *MATERIALS AND METHODS section for the first time.*

**Authors:** We thank the reviewer’s kind suggestion. We have added the description “The myocardial specific caspase-1 was overexpressed *in vivo* by using an adeno associated virus type 9 (AAV9) vector” in MATERIALS AND METHODS section.

*4. Please note the* *internal reference of some* *cleavage of factors, like cleaved caspase-1, -11 and other factors in WB section in figure legends. Are they relative to the GAPDH or their precursors?*

**Authors:** We apologize for the confusion of these WB results. The relative level of these cleaved factors is relative to the GAPDH and we have illustrated their internal reference in the figure legends in our revised manuscript.

*5. There are some misunderstandings and spelling mistakes in this manuscript.*

*Line 138, please offer* *the full name of ‘dp/dt’.*

*Line 213, the word ‘show’ should be changed to ‘showed’.*

*Line 249, the word ‘mediates’ should be changed to ‘mediate’.*

**Authors:** We appreciate the reviewer’s so careful and responsible work for pointing out these mistakes. We have added the full name of ‘dp/dt’ and corrected these grammatical mistakes in our revised manuscript.

**Check List**

***Reviewer 1:***

*Is the English language of sufficient quality?*

*- Yes*

*Is the quality of the figures and tables satisfactory?*

*- Yes*

*Does the reference list cover the relevant literature adequately and in an unbiased manner? - Yes*

*Are the statistical methods valid and correctly applied? (e.g. sample size, choice of test)*

*- Yes*

*Are the methods sufficiently documented to allow replication studies?*

*- Yes*

*Are the data underlying the study available in either the article, supplement, or deposited in a repository? (Sequence/expression data, protein/molecule characterizations, annotations, and taxonomy data are required to be deposited in public repositories prior to publication)*

*- Yes*

*Does the study adhere to ethical standards including ethics committee approval and consent procedure?*

*- Yes*

*Have standard biosecurity and institutional safety procedures been adhered to?*

*- Yes*

**Authors:** We thank the reviewer for the encouragement.

**Reviewer 2:**

**Please summarize the main findings of the study.**

***Reviewer 2:*** *The study by Dai et al revealed a previously unrecognized involvement of caspase‐1 in cardiac HP by regulation of IL‐1β and IL‐18, and shed lights on caspase-1 as an antecedent indicator and target for cardiac hypertrophy.*

**Authors:** We appreciate the reviewer’s summarization of the main findings of the study.

**Please highlight the limitations and strengths.**

***Reviewer 2:*** *Statistical analysis should be improved. If possible, the authors could look for a help from a* *statistician.*

**Authors:** We thank the reviewer for giving us this pertinent suggestion. We apologize for the confusion in the Statistical analysis section. As the reviewer suggested, we consulted a statistician and he provided us professional advices. We re-analyzed the data of this study by using the revised statistical analysis and corrected the description of Statistical analysis section. The following is the revised description.

“All continuous data in our study was presented as the mean ± standard error (SE). GraphPad Prism 8 (GraphPad Software, Version 8.01, San Diego, CA, USA) was used for statistical analysis. The normality tests were performed by the Shapiro-Wilk test and all the continuous data met the normal distribution. The difference between groups was analyzed by one-way ANOVA (Only operation treatment) or two-way ANOVA (Operation and virus injection treatment), followed by Student-Newman-Keuls (SNK) test for multiple comparisons. A p value <0.05 was considered statistically significant.”

**Please comment on the methods, results and data interpretation. If there are any objective errors, or if the conclusions are not supported, you should detail your concerns.**

***Reviewer 2:***

*Statistical analysis: “The data in our study was presented as the mean ± standard error (SE)”. All data or continuous data? Please clarify it.*

**Authors:** We apologize for the negligence of data presentation. In fact, all data in our study is continuous data. We have rephrased the description “All continuous data in our study was presented as the mean ± standard error (SE)”, to make it more concise.

*Statistical analysis: “The difference between groups was* *analyzed by one-way ANOVA with Student-Newman-Keuls (SNK) test”. The analyses in figures 4-6 were performed by using one-way ANOVA? Why no**t* *two-way ANOVA? In addition,* *student-Newman-Keuls (SNK) test could be replaced by the* *post hoc* *Tukey tests? Please explain.*

**Authors:** We thank the reviewer for the constructive suggestions. We apologize for the confusion on statistical description. In figures 4-6, the mice were subjected to two treatments (Re-TAC surgery and AAV9 injection), so the difference between groups should be analyzed by two-way ANOVA. We re-analyzed the data in figures 4-6 and received similar statistical results. The statistical description and figure legends relating to statistical method have been revised, please see the revised manuscript for details. As for the multiple comparison procedures (MCP), both student-Newman-Keuls (SNK) and Tukey tests are widely employed for post hoc comparisons. In general, the post hoc Tukey test is required to meet the same sample size, while the SNK test can be performed with the same or different sample size (1-2). We in our study use SNK test. Moreover, we notice all the groups have the same sample size (n=4), so it is also feasible to use the post hoc Tukey test. We also performed the post hoc Tukey tests and drew similar conclusions on the significance (data not shown).

*[1] Lee, S., and Lee, D.K. (2018). What is the proper way to apply the multiple comparison test? Korean J Anesthesiol 71(5), 353-360. doi: 10.4097/kja.d.18.00242.*

*[2] David S, Phillips. Basic Statistics for Health Science Students. San Francisco: W.H. Frecman and Company, 1978*

*Statistical analysis: For multiple comparisons, if all the variables were not normally distributed, the Kruskal-Wallis test should be used. If not, were all your data normally distributed? Please provide the specific method of statistical analysis* *in the tables and figures.*

**Authors:** We apologize for the omission of normal distribution description. We performed the Shapiro-Wilk test and concluded all the continuous data met the normal distribution. We have added relevant contents on normality test in the Statistical analysis section and on the specific statistical methods we use in the figure legends, pleased see the revised manuscript for details.

**Check List**

***Reviewer 2:***

*Is the English language of sufficient quality?*

*- Yes*

*Is the quality of the figures and tables satisfactory?*

*- Yes*

*Does the reference list cover the relevant literature adequately and in an unbiased manner?*

*- Yes*

*Are the statistical methods valid and correctly applied? (e.g. sample size, choice of test)*

*- Yes*

*Are the methods sufficiently documented to allow replication studies?*

*- No*

*Are the data underlying the study available in either the article, supplement, or deposited in a repository? (Sequence/expression data, protein/molecule characterizations, annotations, and taxonomy data are required to be deposited in public repositories prior to publication)*

*- Yes*

*Does the study adhere to ethical standards including ethics committee approval and consent procedure?*

*- Yes*

*Have standard biosecurity and institutional safety procedures been adhered to?*

*- Yes*

**Authors:** We thank the reviewer for the encouragement and reminders. We have rephrased relevant description to make our methods sufficiently documented to allow replication studies.

**Please provide your detailed review report to the editor and authors (including any comments on the Q4 Check List):**

***Reviewer 2:***

*Statistical analysis: “The data in our study was presented as the mean ± standard error (SE)”. All data or continuous data? Please clarify it.*

*Statistical analysis: “The difference between groups was analyzed by one-way ANOVA with Student-Newman-Keuls (SNK) test”. The analyses in figures 4-6 were performed by using one-way ANOVA? Why not two-way ANOVA? In addition, student-Newman-Keuls (SNK) test could be replaced by the post hoc Tukey tests? Please explain.*

*Statistical analysis: For multiple comparisons, if all the variables were not normally distributed, the Kruskal-Wallis test should be used. If not, were all your data normally distributed? Please provide the specific method of statistical analysis in the tables and figures.*

**Authors:** *Please refer to our response in Q3*

**Reviewer 2**

Q1 Please provide the information of statistician in ”Acknowledgments”

**Authors:** We appreciate the reviewer’s reminder. We are always grateful to the statistician’ s generous contribution to improve this study. We have added the information of statistician in “Acknowledgments” in our revised manuscript.

Q2 All the continuous data met the normal distribution? Sometimes it's hard to do that. If you could, please upload the analyzed data for reproducibility. Pleasure to check together.

**Authors:** We thank the reviewer for the kind and constructive suggestion to help us further improve the manuscript. Inspired by the suggestion, we scrutinized our analyzed data again and performed the Shapiro-Wilk test by three authors (F.D., Xuan L., Xia L.) independently and found the data of LVEDD in Sham group in Figure 1K were not normally distributed. For the data in this group, we used *Kruskal-Wallis test followed by Dunn's test for* multiple comparisons and we have rehearsed the statistical description in our revised manuscript. In addition, although the raw ratios of intensity values relative to GAPDH in Western blot meet the normal distribution, these data in control group show “Invalid input data” in the Shapiro-Wilk test, after they were normalized into “1” to evaluate the protein relative expression level. In order to perform normality test, we reconsidered the homogenization method in WB. After the raw ratios of intensity values relative to GAPDH in all groups were divided by mean of control group, these continuous data in group can be presented as the mean ± standard error, thus these data can be checked by a normality test. We have revised these statistical graphs and reperformed statistical analysis in our revised manuscript. We apologize for our careless and thoughtless description in our last response to the reviewer’s comments. We have uploaded all the analyzed data as attachments for the reviewer’s check.
